# Patterns and Predictors of First-Line Taxane Use in Patients with Metastatic Triple-Negative Breast Cancer in US Clinical Practice

**DOI:** 10.3390/curroncol28040239

**Published:** 2021-07-17

**Authors:** Joyce O’Shaughnessy, Leisha A. Emens, Stephen Y. Chui, Wei Wang, Kenneth Russell, Shih-Wen Lin, Carlos Flores Avile, Patricia Luhn, Andreas Schneeweiss

**Affiliations:** 1 Baylor University Medical Center, Texas Oncology, US Oncology, Dallas, TX 75251, USA; Joyce.OShaughnessy@USONCOLOGY.COM; 2 UPMC Hillman Cancer Center, Magee-Womens Hospital, Pittsburgh, PA 15232, USA; emensla@upmc.edu; 3 Genentech, Inc., South San Francisco, CA 94080, USA; chui.stephen@gene.com (S.Y.C.); lin.shihwen@gene.com (S.-W.L.); 4 Pharmaceutical Outcomes and Policy, University of Florida, Gainesville, FL 32610, USA; vivianwang@ufl.edu; 5 F. Hoffmann-La Roche Ltd., 4070 Basel, Switzerland; kenneth.russell@roche.com; 6 Genesis Research, Hoboken, NJ 07030, USA; carlos@genesisrg.com; 7 National Center for Tumor Disease, Heidelberg University Hospital and German Cancer Research, 69120 Heidelberg, Germany; andreas.schneeweiss@med.uni-heidelberg.de

**Keywords:** metastatic triple-negative breast cancer, chemotherapy, paclitaxel, *nab*-paclitaxel, docetaxel

## Abstract

We investigated first-line (1L) treatment patterns and predictors of taxane use to better understand the evolving metastatic triple-negative breast cancer (mTNBC) treatment landscape. This retrospective analysis of the Truven Health MarketScan^®^ (Somers, NY, USA) Database included women with mTNBC who received 1L therapy within six months of diagnosis (January 2005–June 2015). Multivariate logistic regression models identified predictors of taxane use, adjusting for prognostic factors. A total of 2271 women with newly diagnosed mTNBC received 1L treatment during the study period. Half received a 1L taxane (53%), more often in combination than as monotherapy (58% versus 42%), though this varied by specific taxane. *Nab*-Paclitaxel monotherapy increased substantially after 2010. More recent treatment year (odds ratio, 2.16 (95% CI 1.69–2.76]) and number of metastases (≥3 versus 1: 1.73 (1.25–2.40)) predicted taxane monotherapy versus combination. Having a health maintenance organization versus a preferred provider organization plan predicted less *nab*-paclitaxel versus paclitaxel (0.32 (0.13–0.80)) or docetaxel (0.30 (0.10–0.89)) use. More recent index year (2011–2015 vs. 2005–2010) was the only predictor favoring *nab*-paclitaxel versus paclitaxel (2.01 (1.26–3.21)) or docetaxel (3.63 (2.11–6.26)). Taxane-containing regimens remained the most common 1L mTNBC treatments. Paclitaxel and *nab*-paclitaxel use changed substantially over time, with *nab*-paclitaxel use associated with insurance coverage.

## 1. Introduction

Metastatic triple-negative breast cancer (mTNBC) comprises 15% to 20% of all breast cancer diagnoses [1]. Patients with mTNBC tend to be diagnosed at a younger age and have a worse prognosis than those with other subtypes of breast cancer [2,3,4]. A chart review study suggested that most, but not all, patients with mTNBC in US community practices receive a systemic treatment (83%); the observed progression-free survival (PFS) of these treated patients was approximately four months [5]. Real-world overall survival estimates range from 8 to 17 months [3,5,6]. International treatment guidelines have historically recommended taxanes and anthracyclines (for patients previously not exposed to anthracyclines) as the foundation of first-line (1L) chemotherapy for patients with mTNBC [7,8]. 

Taxanes combined with anti–programmed death-ligand 1/programmed death-1 (anti–PD-L1/PD-1) immune checkpoint inhibitors may improve anti-tumor responses in some patients, and are being explored in ongoing clinical studies [9,10,11]. The IMpassion130 study was the first phase three study to demonstrate the benefit of immunotherapy combined with chemotherapy in mTNBC. Atezolizumab plus nanoparticle albumin-bound paclitaxel (*nab*-paclitaxel) provided a significant PFS benefit and clinically meaningful overall survival improvement in patients with PD-L1+ mTNBC, compared with placebo plus *nab*-paclitaxel [12,13]. As such, atezolizumab combined with *nab*-paclitaxel is recommended for the treatment of patients with PD-L1+ mTNBC [14,15]. Another immune checkpoint inhibitor, pembrolizumab, has reported a PFS benefit in combination with chemotherapy (*nab*-paclitaxel, paclitaxel, or gemcitabine/carboplatin) for 1L treatment of mTNBC [11], and has been granted US Food and Drug administration approval in PD-L1+ patients.

With the introduction of new combination regimens for mTNBC, including a taxane plus cancer immunotherapy for mTNBC, there is a need to better understand the evolving use of 1L taxanes for mTNBC in clinical practice, to inform clinical and policy decisions. 

This analysis was conducted to examine 1L mTNBC treatment patterns, with a focus on taxane chemotherapy. We also evaluated the characteristics of patients receiving taxanes, and factors that might predict the use of taxane-based regimens for 1L treatment of mTNBC in US clinical practice.

## 2. Results

### 2.1. Patient Characteristics

A total of 2271 female patients with newly diagnosed mTNBC received 1L treatment during the study period, and were included in the analysis. The median age at treatment initiation was 58 years; most patients lived in metropolitan areas (83%) and were covered by commercial health insurance (77%; Table 1). Across treatment groups, patients were otherwise generally healthy, with most having a CCI score of 0 (82–87%). Common sites of metastases were bone (47–61%), lungs (30–36%), and liver (22–34%; Table 1).

### 2.2. 1L Taxane Treatment Patterns

Slightly more than half of patients received a taxane-containing 1L treatment regimen during the study period (1208/2271, 53%); nearly half received a paclitaxel-containing regimen (579/1208, 48%), 31% docetaxel (370/1208), and 21% *nab*-paclitaxel (259/1208; Table 2). Overall, nearly half (574/1208, 48%) of patients treated with taxane-containing regimens received weekly administrations, which varied by specific taxane. First-line paclitaxel- and *nab*-paclitaxel-containing regimens were most often given weekly (to 58% and 67% of these patients, respectively), and 1L docetaxel-containing regimens were typically given every 3 weeks (71%).

**Figure 1:**
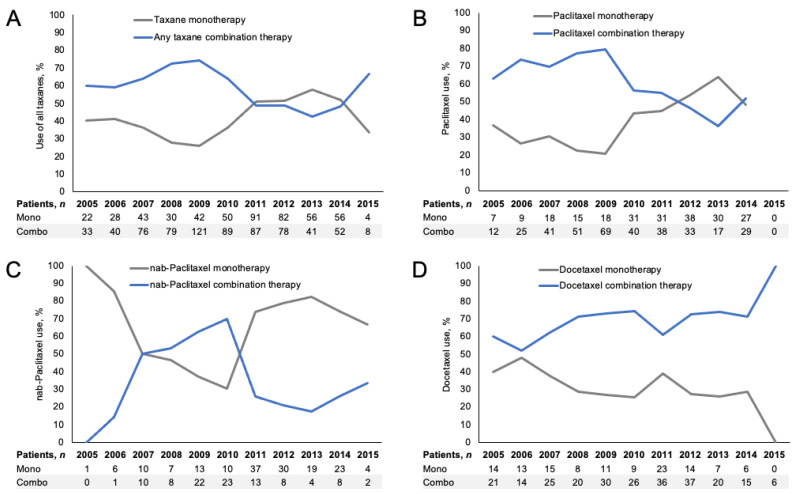
Use of taxane monotherapy and combination therapy in 1L mTNBC by year (2005–2015): (**A**) all taxanes, (**B**) paclitaxel, (**C**) *nab*-paclitaxel, and (**D**) docetaxel. Proportions are plotted, with absolute numbers shown below the plots. At the time of the analysis, treatment use data were available only through 30 June 2015. *1L:* first line, *mTNBC:* metastatic triple-negative breast cancer.

More patients received 1L taxane treatment as part of a combination regimen (704/1208, 58%) than as monotherapy (504/1208, 42%), although this varied by the specific taxane. *Nab*-paclitaxel was most often used as a monotherapy after 2010, rather than as a combination therapy (Figure 1). Paclitaxel was generally used more often in combination regimens prior to 2012, after which combination and monotherapy appeared to be used in similar proportions. The slight increase in aggregated taxane monotherapy use after 2010 may have been driven by the increased use of *nab*-paclitaxel monotherapy, and decreased use of paclitaxel in combination therapy. Docetaxel was predominantly used in combination therapy throughout the entire study period, with no notable trends or changes.

### 2.3. Predictors of 1L mTNBC Taxane Use

**Figure 2:**
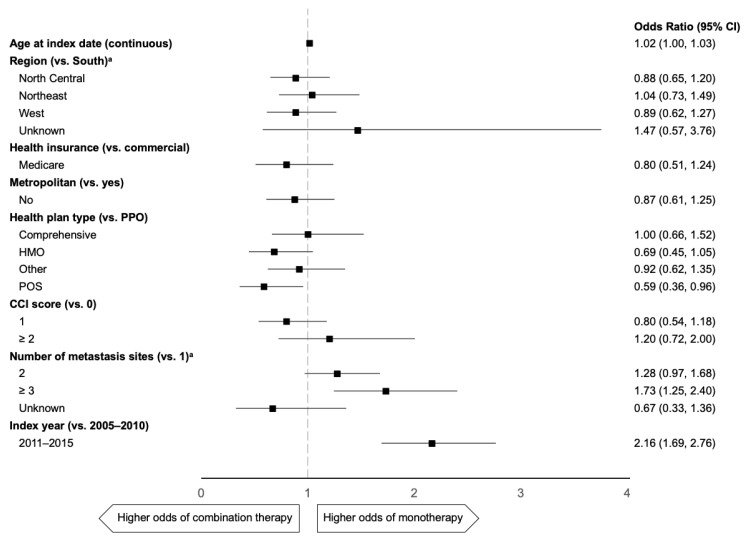
Predictors of taxane monotherapy versus combination therapy in 1L mTNBC. 1L: first line, CCI: Charlson Comorbidity Index, HMO: health maintenance organization, mTNBC: metastatic triple-negative breast cancer, POS: point of service, PPO: preferred provider organization. ^a^ Unknown categories are not shown because of unstable estimates from small sample sizes.

Across all taxane-containing regimens, receiving a taxane as monotherapy rather than as combination therapy was predicted by a more recent index year of treatment (odds ratio (OR), 2.16 (95% CI 1.69–2.76)) and number of metastases (≥3 versus 1: OR, 1.73 (95% CI 1.25–2.40); Figure 2). Patients with a point of service (POS) health plan were less likely to receive a taxane as monotherapy than in combination versus those with a preferred provider organization (PPO) plan (OR, 0.59 (95% CI 0.36–0.96)).

**Figure 3:**
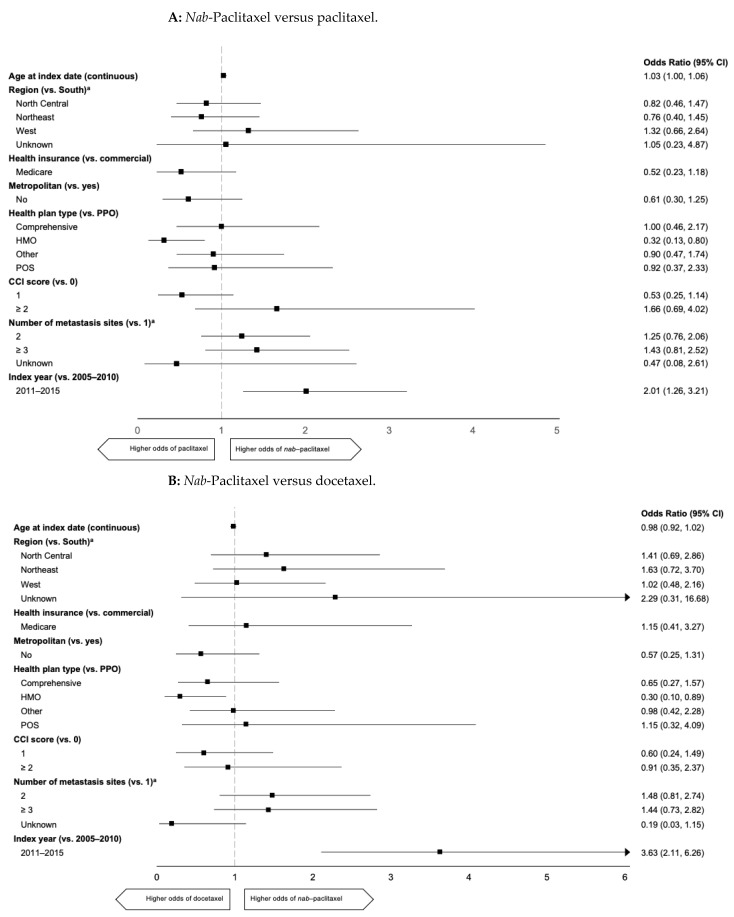

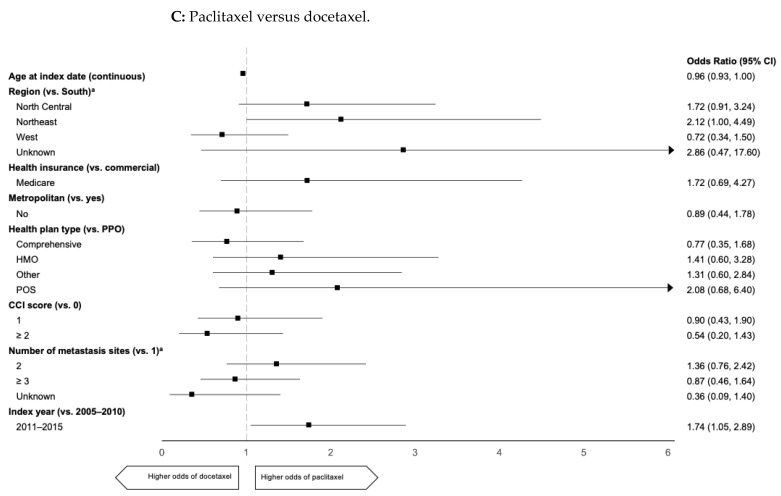
Predictors of specific taxanes used in 1L mTNBC. (**A**) *nab*-Paclitaxel versus paclitaxel. 1L: first line, CCI: Charlson Comorbidity Index, HMO: health maintenance organization, mTNBC metastatic triple-negative breast cancer, POS: point of service, PPO: preferred provider organization. Arrowheads indicate a CI wider than the *x* axis. ^a^ Unknown categories are not shown because of unstable estimates from small sample sizes.

For use of a specific taxane over another, predictors included more recent index year of treatment and type of insurance coverage (Figure 3). Patients with a HMO plan were less likely than those with a PPO plan to receive *nab*-paclitaxel compared with paclitaxel (OR, 0.32 (95% CI 0.13–0.80)) or docetaxel (OR, 0.30 (95% CI 0.10–0.89)). Patients with a more recent index treatment year were also more likely to receive *nab*-paclitaxel than paclitaxel (OR, 2.01 (95% CI 1.26–3.21)) or docetaxel (OR, 3.63 (95% CI 2.11–6.26)), and were more likely to receive paclitaxel than docetaxel (OR, 1.74 (95% CI 1.05–2.89)). No differences based on geographic region or CCI score were observed (Figure 3).

## 3. Discussion

This study has illustrated 1L treatment patterns for patients with newly diagnosed mTNBC in US clinical practice. Taxane-containing regimens were the most common 1L treatment for mTNBC between 2005 and 2015 (53%), and we observed a notable uptake in *nab*-paclitaxel monotherapy after 2010. Concerning the taxanes, observable patient characteristics were similar among those who received *nab*-paclitaxel versus paclitaxel, with the exception of health plan coverage (patients with a health maintenance organization (HMO) plan were more likely than patients with a PPO plan to receive paclitaxel) and having a more recent index treatment year (favoring use of *nab*-paclitaxel). The difference based on type of health plan suggests that these agents may be prescribed interchangeably when insurance coverage is not a factor.

Our observations of real-world treatment practices may be viewed in the context of recent studies of 1L taxane use for mTNBC. In our study, only two variables—type of commercial health plan coverage and more recent index treatment year—were significant predictors of receiving *nab*-paclitaxel versus paclitaxel treatment. A 2011–2016 health record study showed that patients who received 1L *nab*-paclitaxel monotherapy tended to have an earlier-stage disease at diagnosis, recurrent disease, prior adjuvant taxane treatment, and prior neuropathy than those who received 1L paclitaxel [16]. Commercial insurance coverage tended to be higher in patients who received *nab*-paclitaxel, but the differences were not statistically significant. Also, in this current study, a substantial increase in *nab*-paclitaxel monotherapy use was seen after 2010, which could have been related to the improved PFS reported with *nab*-paclitaxel versus paclitaxel treatment in the phase 2 clinical program for patients with metastatic breast cancer [17]. Further investigation is required to thoroughly examine the potential reasons for this observation, given more recent data and real-world findings on the use of *nab*-paclitaxel vs. paclitaxel for metastatic breast cancer [16,18,19,20].

Our real-world study observed that an increased disease burden (≥3 sites of metastases) predicted receipt of 1L taxane treatment as monotherapy rather than in a combination regimen. This observation does not appear to have substantial context in the scientific literature. Furthermore, treatment guidelines (e.g., [14,15]) tend to recommend sequential single-agent regimens for HER2-negative disease, with combination chemotherapy being used for select cases, including patients with high tumor burden, rapid disease progression, or visceral crisis. It is possible that this observation in our study could have been indicative of a small sample size among those who received monotherapy (42% of taxanes were given as monotherapy, and only 17% of all patients had ≥3 metastatic sites). A targeted investigation is warranted to further explore this interesting finding and any related contributors (e.g., influences by type of metastatic site, comorbidities affecting tolerability).

This study should be interpreted in the context of certain strengths and limitations. The nature of the data set (coding for administrative purposes) may not fully represent the clinical circumstances encountered and considered by the health care provider, and more detailed clinical information from medical records was not available. Whether patients were treated in academic or community cancer centers was unknown, and may have contributed to regional differences in treatment patterns. This study examined reimbursed 1L mTNBC treatment patterns over a 10-year period based on real-world data, which cannot be realistically evaluated in a clinical trial. These findings may not be generalizable to patients not covered by commercial or Medicare insurance (such as Medicaid or other plans) or to those treated outside the United States, due to variability in health systems, healthcare payment models, and clinical practices. Though *nab*-paclitaxel is rarely used in Europe, increasing access to atezolizumab by national insurance systems in EU countries may lead to wider use of *nab*-paclitaxel as the only approved partner of the PD-L1 inhibitor. These findings may inform clinical trial design and population health management decisions, because the use of immune checkpoint inhibitors and other targeted agents alone and in combination with chemotherapy is prevalent and may continue to grow.

The use of taxanes in the 1L treatment of women with newly diagnosed mTNBC has changed over the past 10 years, with a notable increase in the use of *nab*-paclitaxel as monotherapy. Type of health insurance plan, indicators of disease burden, and geography, all appear to contribute to taxane selection in 1L treatment. The observed variability in specific taxane use for 1L treatment may be explored in future research, including any further impact that insurance coverage may have on treatment selection and outcomes for women with mTNBC. The use of a 1L regimen combining a taxane with a new innovative treatment option may offer promising prospects for research and treatment of women with mTNBC.

## 4. Materials and Methods

### 4.1. Data Source

We conducted a retrospective analysis of administrative claims using the Truven Health MarketScan^®^ (now IBM^®^ MarketScan^®^) Commercial and Medicare Supplemental Databases (Truven Health Analytics (now IBM Watson Health), Ann Arbor, MI, USA). The Truven MarketScan data set includes commercial insurance and Medicare Supplemental claims for medical encounters and prescriptions from >180 million individuals in the United States. All beneficiary records were de-identified in compliance with applicable protections and regulations.

### 4.2. Patient Population

Patients were indexed based on the first claim in their record for a treatment of interest (Supplementary Table S1). Further inclusion criteria included female sex; age ≥ 18 years at treatment initiation; treatment initiation date (index date) between 1 January 2005 and 30 June 2015; ≥1 year of enrollment in the patient’s health plan prior to the index date (30-day gap allowed); ≥2 claims for primary breast cancer (International Classification of Diseases, Ninth Revision, Clinical Modification (ICD-9-CM):174.x) within 30 days of each other and ≥1 day apart during the 12 months prior to the index date; ≥2 claims ≥1 day apart for secondary malignancies (ICD-9-CM: 196.0, 196.1, 196.2, 196.5, 196.6, 196.8, 197.x-198.x (except 198.2 and 198.81)) on or before the index date, the first of which was considered the mTNBC diagnosis date and must have occurred within the 6 months prior to the index date; and ≥1 claim for estrogen receptor/progesterone receptor/human epidermal growth factor receptor 2 (HER2) immunohistochemistry testing (Current Procedural Terminology code: 88360) or ≥1 claim for HER2 fluorescence in situ hybridization testing (Current Procedural Terminology codes: 88271, 88274, 88367, 88368, 88369) at any time during the patient’s enrollment. Patients were excluded if they met any of the following criteria: ≥1 claim for a hormonal treatment or HER2-targeted therapy or ≥1 claim for estrogen receptor positive status (ICD-9-CM: V86.0) at any time during their enrollment and ≥2 claims for the same ICD-9-CM codes for another primary malignancy within 30 days of each other and ≥1 days apart (ICD-9-CM: 140.xx-208.22, except 174.x and 196.x-198.x) during the six months prior to their mTNBC diagnosis date.

### 4.3. Exposure Definition

The timing of 1L treatment was defined by the “start” and “stop” of 1L mTNBC treatment, where “treatment start” was the date of the first mTNBC chemotherapy administration (determined from a list of known treatments used for TNBC). “Treatment stop” was defined as a break in treatment of ≥180 days, initiation of a new mTNBC treatment > 30 days after first treatment start, or discontinuation of all drugs in a combination regimen. Exceptions were made for treatment changes, including substitution of cisplatin for carboplatin (or vice versa) in a platinum combination regimen or substitution of a different taxane in a taxane-containing regimen.

Monotherapy was defined as a claim for only one type of anti-cancer drug, and combination therapy was defined as a claim for >1 type of anti-cancer drug within 30 days of mTNBC treatment initiation. The treatment schedule was determined by the number of taxane administrations in the first 60 days of treatment (or until the end of 1L, whichever came first): weekly (5+ administrations; this included patients who received administrations every 3 out of 4 weeks), every 3 weeks (3–4 administrations), or “other” (1–2 administrations).

### 4.4. Covariate Definitions

To be considered to have metastases to a specific site, a patient was required to have ≥2 claims for secondary malignancies in the same organ within the six months prior to and up to two months after the patient’s index date. The organ groupings were as follows: bone (ICD-9-CM: 198.5), brain (ICD-9-CM: 198.3-198.4), liver (ICD-9-CM: 197.7), lung (ICD-9-CM: 198.0–197.3), and other (ICD-9-CM: 196.0, 196.1, 196.2, 196.5, 196.6, 196.8, 197.4–197.6, 197.8, 198.0, 198.6, 198.7, 198.82, 198.89). If a patient had only one claim for a code or given organ site during the time window, they were considered uncategorized/unknown.

Comorbidity burden was quantified using a modified and updated version of the Charlson Comorbidity Index (CCI) [21,22,23]. All components of the CCI were included except metastatic cancer, because all patients had this condition. The individual conditions as well as associated ICD-9-CM codes are listed in the Supplementary Table S2. A patient was considered to have a given condition if they had ≥1 inpatient claim or ≥2 outpatient claims for the same condition that occurred in the 12 months prior to the index date, inclusive. 

### 4.5. Statistical Analysis

Descriptive statistics were used to summarize patient demographic and clinical characteristics and 1L mTNBC treatment. Continuous variables were summarized with medians and interquartile ranges; categorical variables were summarized with counts and percentages. Differences in distributions were examined using a chi-square or Student *t* test, as appropriate. Multivariate logistic regression models were used to identify independent predictors of taxane use, evaluating associations between each taxane monotherapy and taxane monotherapy versus combination therapy. Models adjusted for key prognostic factors at the start of 1L chemotherapy, including age (continuous years), region (Northeast, North Central, South, West, unknown), urbanicity (metropolitan, rural), health insurance (commercial, Medicare), health plan (comprehensive, HMO, POS, PPO, other), CCI (0, 1, 2+), number of metastatic sites (1, 2, 3+), and calendar year (categorical; 2005–2010 versus 2011–2015). All analyses were conducted using SAS Studio version 9.04 or higher.

## Figures and Tables

**Table 1:** Patient demographic and clinical characteristics.

Characteristic	All Patients (*n* = 2271)	1L Paclitaxel (*n* = 579)	1L *nab*-Paclitaxel (*n* = 259)	1L Docetaxel (*n* = 370)
Age, median (IQR), years	58 (51–64)	58 (51–65)	59 (53–64)	59 (53–65)
Metropolitan area, *n* (%)	1895 (83)	480 (83)	226 (87)	319 (86)
US region, *n* (%)				
Northeast	447 (20)	101 (17)	50 (19)	50 (13)
North Central	585 (26)	165 (29)	65 (25)	81 (22)
South	842 (37)	219 (38)	94 (36)	166 (45)
West	344 (15)	83 (14)	44 (17)	67 (18)
Unknown	53 (2)	11 (2)	6 (2)	6 (2)
Health insurance, *n* (%)				
Commercial coverage	1751 (77)	442 (76)	197 (76)	289 (78)
Medicare	520 (23)	137 (24)	62 (24)	81 (22)
Health plan type, *n* (%)				
Comprehensive	280 (12)	75 (13)	32 (12)	52 (14)
HMO	223 (10)	58 (10)	12 (5)	48 (13)
PPO	1347 (59)	347 (60)	157 (61)	199 (54)
POS	166 (7)	41 (7)	23 (9)	28 (8)
Other	255 (11)	58 (10)	35 (13)	43 (12)
Charlson Comorbidity Index, *n* (%)				
0	1903 (84)	483 (83)	224 (87)	302 (82)
1	245 (11)	70 (12)	16 (16)	43 (12)
≥2	123 (5)	26 (5)	19 (7)	25 (7)
1L treatment index date, *n* (%)				
2005–2010	1142 (50)	336 (58)	111 (43)	206 (56)
2011–2015	1129 (50)	243 (42)	148 (57)	164 (44)
Site of metastases, *n* (%)				
*Location*				
Bone	1089 (48)	296 (51)	157 (61)	174 (47)
Brain	311 (14)	68 (12)	33 (13)	54 (15)
Liver	599 (26)	174 (30)	89 (34)	82 (22)
Lung	743 (33)	208 (36)	86 (33)	111 (30)
Other	1008 (44)	273 (47)	107 (41)	144 (39)
Number of unique organ sites of metastases, *n* (%)				
1	1103 (49)	269 (47)	102 (39)	207 (56)
2	670 (29)	183 (32)	103 (40)	88 (24)
≥3	388 (17)	111 (19)	50 (19)	54 (15)
Unknown	110 (5)	16 (3)	4 (2)	21 (6)

1L: first line, HMO: health maintenance organization, IQR: interquartile range, POS: point of service, PPO: preferred provider organization Taxanes could have been used as 1L monotherapy or as part of 1L combination therapy. All values may not total to 100% because of rounding.

**Table 2:** Taxanes in 1L mTNBC during the study period (January 2005 through June 2015).

Treatment Regimen, *n* (%)	Any Taxane Regimen (*n* = 1208)	1L Paclitaxel (*n* = 579)	1L *nab*-Paclitaxel (*n* = 259)	1L Docetaxel (*n* = 370)
Monotherapy	504 (42)	224 (39)	160 (62)	120 (32)
Doublet	566 (47)	302 (52)	89 (34)	175 (47)
+ platinum	203 (36)	137 (45)	19 (21)	47 (27)
+ bevacizumab ^a^	192 (34)	122 (40)	58 (65)	12 (7)
+ anthracycline	14 (2)	5 (2)	2 (2)	7 (4)
+ other agent	157 (28)	38 (13)	10 (11)	109 (62)
Triplet or more	138 (11)	53 (9)	10 (4)	75 (20)
Schedule for administration				
qw	574 (48)	338 (58)	175 (67)	61 (16)
q3w	518 (43)	195 (34)	60 (23)	263 (71)
Other	116 (10)	46 (8)	24 (9)	46 (12)

1L: first line, q3w: every 3 weeks, qw: weekly regimens (includes every 3 out of 4 week schedules), other did not fit into other 2 categories, including patients with single administrations for the given drug. Taxanes could have been used as 1L monotherapy or as part of 1L combination therapy. Components of any taxane regimen do not total to 100% because of rounding; boldface rows sum to 100% of all treatment regimens; types of doublet regimens sum to 100% of doublet therapy regimens. ^a^ Most patients (188/192, 98%) were treated before the breast cancer indication was removed for bevacizumab in November 2011.

## Data Availability

The data that support the findings of this study are available from Truven Health MarketScan^®^ Commercial and Medicare Supplemental Databases (Truven Health Analytics, Ann Arbor, MI, USA), but restrictions apply to the availability of these data, which were used under license for the current study, and so are not publicly available. Data are, however, available from the authors upon reasonable request and with permission of Truven Health MarketScan^®^.

## Supplementary Materials

The following are available online at https://www.mdpi.com/article/10.3390/curroncol28040239/s1, Table S1: Included treatments of interest, Table S2: Charlson Comorbidity Index conditions and coding.
