# Supplementary material for: Patterns and Predictors of First-Line Taxane Use in Patients with Metastatic Triple-Negative Breast Cancer in US Clinical Practice
Source: Curr Oncol. 2021 Jul 17;28(4):2741–52. doi: 10.3390/curroncol28040239 (PMC8293053; doi:10.3390/curroncol28040239)
Supplement: Supplementary file 1 [file curroncol-28-00239-s001.zip › curroncol-1279986-supplementary.pdf]

Current Oncology

MDPI

Article

Patterns and Predictors of First-Line Taxane Use in Patients with Metastatic Triple-Negative Breast Cancer in US Clinical Practice

Joyce O'Shaughnessy [1], Leisha A. Emens [2], Stephen Y. Chui [3], Wei Wang [4,a], Kenneth Russell [5], Shih-Wen Lin [3], Carlos Flores Avile [6], Patricia Luhn [3,*] and Andreas Schneeweiss [7]

SUPPLEMENTARY MATERIALS

Supplementary Table 1 Included treatments of interest

Drug Name	HCPCS and NDCs Included
**Taxanes**	
Paclitaxel (Nov-Onxol, Taxol)	C9431, J9265
Docetaxel (Novaplus, Taxotere)	J9170, J9171
nab-Paclitaxel (Abraxane)	C9127, J9264
**Anthracyclines**	
Doxorubicin (Doxil)	C9415, J9000, J9001, Q2050, Q2049, J9002, Q2048
Epirubicin	J9178
**Platinum agents**	
Carboplatin (Amerinet, Paraplatin)	J9045
Cisplatin	C9418, J9060, J9062
**Targeted therapies**	
Bevacizumab (Avastin)	C9214, C9257, J9035, Q2024, S0116
Cetuximab	C9215, J9055
**Antimetabolites**	
Gemcitabine (Gemzar)	J9201
Capecitabine (Xeloda)	J8520, J8521
5-Fluorouracil	J9190
Methotrexate	J8610, J9250, J9260
**Alkylating agents**	
Cyclophosphamide	C9420, C9421, J8530, J9070, J9080, J9090, J9091, J9092, J9093, J9094, J9095, J9096, J9097
Temozolomide	C1086, C9253, J8700, J9328
**Antimicrotubular agents**	
Vinorelbine	J9390, C9440
Ixabepilone	C9240, J9207
Eribulin mesylate	C9280, J9179
**HER2 agents**	
Trastuzumab (Herceptin)	J9355, 50242013460, 50242005656, 50242013468

**Drug Name**	**HCPCS and NDCs Included**
Pertuzumab (Perjeta)	C9292, J9306, 50242014501
Ado-trastuzumab emtansine (Kadcyla)	J9354, C9131, 50242008801, 50242008701
Lapatinib	173075200
**Hormonal therapies**	
Any selective estrogen receptor modulator (SERM)	V07.51
Any aromatase inhibitor (AI)	V07.52
Anastrazole (Arimidex)	S0170, 51991062010, 54569619800, 378603477, 16729003510, 66336053390, 63275993003, 38779255504, 63275993002, 33261095700, 62033037606, 38779227403, 62756025083, 68001015508, 67877017110, 51079032306, 93753656, 38779227404, 33261095790, 54016413, 68382020906, 51079032301, 60429028690, 62756025013, 66336053330, 51927443500, 33261095730, 378603405, 51655063853, 63275993004, 38779255503, 38779227406, 68084044821, 60505298503, 46144020005, 904622961, 42043018003, 378603493, 60258086603, 42254016130, 16571042103, 42291010530, 63275993001, 16729003516, 33261095760, 60429028630, 68001015504, 55111064730, 68382020910, 54868613001, 54868613000, 51991062033, 76418001301, 21695099030, 67877017130, 904619546, 781535631, 63323012930, 16729003515, 63275993005, 38779255506, 46144020001, 66435041530, 60687011211, 60687011221, 54569573100, 310020137, 54868500000, 310020130, 55175550503, 12280034630, 35356027030
Exemestane (Aromasin)	S0156, 63629126201, 54569573200, 49999098630, 54868526100, 9766304, 60687013221, 54008013, 47781010830, 59762285801, 60687013211
Fulvestrant (Faslodex)	J9395, 310072010, 310072050, 310072025,
Lapatinib (Tykerb)	173075200
Letrozole (Femara)	35356040930, 78024915, 54868415100, 54569571400, 60505325508, 51991075933, 68258595503, 16729003415, 63323077230, 54026913, 55111064630, 603418016, 60505325503, 53217010830, 62756051183, 378207105, 16729003410, 68084080311, 378207193, 68084080321, 54868625200, 42291037490, 42254024330, 93762056, 51991075910
Tamoxifen (Nolvadex, Soltamox, Novoclair, Nuvya)	S0187, 310060412, 57866661501, 55175550006, 403150571, 310060025, 66105083201, 58016065760, 66105083210, 54569038202, 54569853100, 310060018, 55289058530, 60346004832, 310060075, 310060430, 66105083203, 57866661801, 66105083209, 66105083206, 54569038200, 310060060, 310060490, 13632012301, 63370025115, 310073130, 54868300402, 62991115101, 38779034110, 54868300405, 49452757102, 38779034180, 591223319, 38779034103, 49452775301, 54868300401, 52372075602, 63307047005, 49452757104, 591223330, 172565658, 172565746, 38779034101, 172565780, 555090401, 51927297600, 172565760, 93078486, 68258596006, 63370025110, 62991115103, 54483413, 63307047001, 63370025135, 49452775302, 54883125, 172565770, 555044663, 62991115102, 54569860200, 54883425, 52372075601, 54868428703, 75840011001, 38779034104, 93078205, 54483422, 38779034108, 60346090060, 42254034390, 62991115104, 38779034130, 63739026915, 93078410, 54569376500, 49452775305,

Drug Name	HCPCS and NDCs Included
	555044609, 172565670, 555090414, 378014491, 591247319, 591247260, 54868428702, 49452757103, 38779034140, 49452775304, 38779034150, 54868428704, 54569376501, 63739026910, 54569585700, 54868428700, 591247330, 555090405, 93078256, 49452757106, 591247218, 310073060, 172565680, 54569571600, 54483121, 38779034105, 93078406, 591223218, 54868300404, 54483126, 49452757105, 555044605, 378027401, 93078405, 63370025125, 54868300403, 54868428701, 378027493, 172565649, 93078201, 51552083802, 555044603, 49452775303, 54868300400, 93078210, 49452757101, 378014405, 591223260, 69167048305, 69167048505
Toremifene (Fareston)	85112601, 11399000530, 42747032730, 54092017030, 54092017001, 85112602, 11399000501

HCPCS Healthcare Common Procedure Coding System, *HER2* human epidermal growth factor receptor 2, *NDC* National Drug Code

Supplementary Table 2 Charlson Comorbidity Index conditions and coding

Comorbidity	ICD-9-CM	Description	Index weight
Chronic pulmonary disease	490-496	Chronic obstructive pulmonary disease	1
	500-505	Pneumoconioses	
	506.4	Chronic respiratory condition due to fumes and vapors	
Diabetes without complications	250-250.3	Diabetes with or without acute metabolic disturbances	0
	250.7	Diabetes with peripheral circulatory disorders	
Diabetes with complications	250.4-250.6	Diabetes with renal, ophthalmic, or neurological manifestations	1
Congestive heart failure	428-428.9	Heart failure	2
Cerebrovascular disease	430-438	Cerebrovascular disease	0
Peripheral vascular disease	443.9	PVD	0
	441.1-441.9	Aortic aneurysm	
	785.4	Gangrene	
	V43.4	Blood vessel replaced by prosthesis	
	Procedure 38.48	Resection and replacement of lower limb arteries	
Acute myocardial infarction	410-410.9	Acute MI	0
Old myocardial infarction	412	Old MI	0
Rheumatologic disease	710	Systemic lupus erythematosus	1
	710.1	Systemic sclerosis	
	710.4	Polymyositis	
	714.0-714.2	Adult rheumatoid arthritis	
	714.81	Rheumatoid lung	

**Comorbidity**	**ICD-9-CM**	**Description**	**Index weight**
	725	Polymyalgia rheumatica	
Moderate/severe renal disease	582.0-582.9	Chronic glomerulonephritis	1
	583.0-583.7	Nephritis and nephropathy	
	585	Chronic renal failure	
	586	Renal failure, unspecified	
	588.0-588.9	Disorders resulting from impaired renal function	
Dementia	290.0-290.9	Senile and presenile dementias	2
Ulcer disease	531.0-534.9	Gastric, duodenal, and gastrojejunal ulcers	0
	531.4-531.7	Chronic forms of peptic ulcer disease (subsets of above)	
	532.4-532.7		
	533.4-533.7		
	534.4-534.7		
Paralysis	344.1	Paraplegia	2
	342.0-342.9	Hemiplegia	
Mild liver disease	571.2	Alcoholic cirrhosis	2
	571.5	Cirrhosis without mention of alcohol	
	571.6	Biliary cirrhosis	
	571.4-571.49	Chronic hepatitis	
Moderate/severe liver disease	572.2-572.8	Hepatic coma, portal hypertension, other sequelae of chronic liver disease	4
	456.0-456.21	Esophageal varices	
AIDS	042-044.9	HIV infection with related specified conditions	4
Neuropathy	357.6	Polyneuropathy due to drugs	NA
	782.0	Disturbance of skin sensation	
	357	INFLAM/TOXIC NEUROPATHY	
	357.0	Acute infective polyneuritis	
	357.1	Polyneuropathy in collagen vascular disease	
	357.2	Polyneuropathy in diabetes	
	357.3	Polyneuropathy in malignant disease	
	357.4	Polyneuropathy in other diseases classified elsewhere	
	357.5	Alcoholic polyneuropathy	
	357.7	Polyneuropathy due to other toxic agents	
	357.8	INFLAM/TOX NEUROPATHY NEC	
	357.81	Chronic inflammatory demyelinating polyneuritis	
	357.82	Critical illness polyneuropathy	
	357.89	Other inflammatory and toxic neuropathy	
	357.9	Unspecified inflammatory and toxic neuropathy	
	377.34	Toxic optic neuropathy	
	377.2	OPTIC DISC DISORDERS NEC	
	377.21	Drusen of optic disc	

**Comorbidity**	**ICD-9-CM**	**Description**	**Index weight**
	377.22	Crater-like holes of optic disc	
	377.23	Coloboma of optic disc	
	377.24	Pseudopapilledema	
	353.4	Lumbosacral root lesions, not elsewhere classified	
	355.7	MONONEURITIS LEG NEC	
	355.71	Causalgia of lower limb	
	355.79	Other mononeuritis of lower limb	
	355.9	Mononeuritis of unspecified site	
	353.0	Brachial plexus lesions	

AIDS acquired immunodeficiency syndrome, *HCPCS* Healthcare Common Procedure Coding System, *HIV* human immunodeficiency virus, *ICD-9-CM* International Classification of Diseases, Ninth Revision, Clinical Modification, *MI* myocardial infraction, *NDC* National Drug Code, *PVD* peripheral vascular disease.
